# Supplementary material for: Genomics Characterization of an Engineered Corynebacterium glutamicum in Bioreactor Cultivation Under Ionic Liquid Stress
Source: Front Bioeng Biotechnol. 2021 Nov 18;9:766674. doi: 10.3389/fbioe.2021.766674 (PMC8637627; doi:10.3389/fbioe.2021.766674)
Supplement: Supplementary file 1 [file DataSheet1.PDF]

Supplementary Materials

Genomics Characterization of an engineered *Corynebacterium glutamicum* in Bioreactor Cultivation under Ionic Liquid Stress

Deepanwita Banerjee [a,d,#], Thomas Eng [a,d,#], Yusuke Sasaki[a,d], Aparajitha Srinivasan [a,d], Asun Oka, Robin A. Herbert, Jessica Trinh, Vasanth R. Singan[b,d], Ning Sun[c,d], Dan Putnam[e], Corinne Scown, Blake Simmons, and Aindrila Mukhopadhyay [a,d]*

[a]Joint BioEnergy Institute, Lawrence Berkeley National Laboratory, Emeryville, CA 94608, USA.
[b]Joint Genome Institute, Lawrence Berkeley National Laboratory, Berkeley, CA 94720, USA.
[c]Advanced Biofuels and Bioproducts Process Development Unit, Lawrence Berkeley National Laboratory, Emeryville, CA 94608, USA.
[d]Biological Systems and Engineering Division, Lawrence Berkeley National Laboratory, Berkeley, CA 94720, USA
[e] UC Davis
# Equal contributors: D. Banerjee, T. Eng
*Corresponding author: Aindrila Mukhopadhyay, amukhopadhyay@lbl.gov

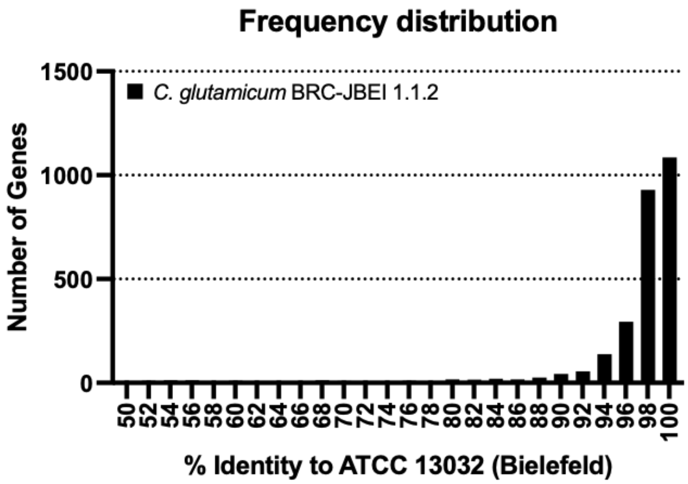

Figure S1: Annotating *C. glutamicum* BRC-JBEI 1.1.2 with characterized genes from the C. glutamicum ATCC 13032 (Bielefeld) assembly. 2,777 Cgl genes were annotated in the *C. glutamicum* BRC-JBEI 1.1.2 assembly. Seven genes are in the histogram bin of 50 % identity.

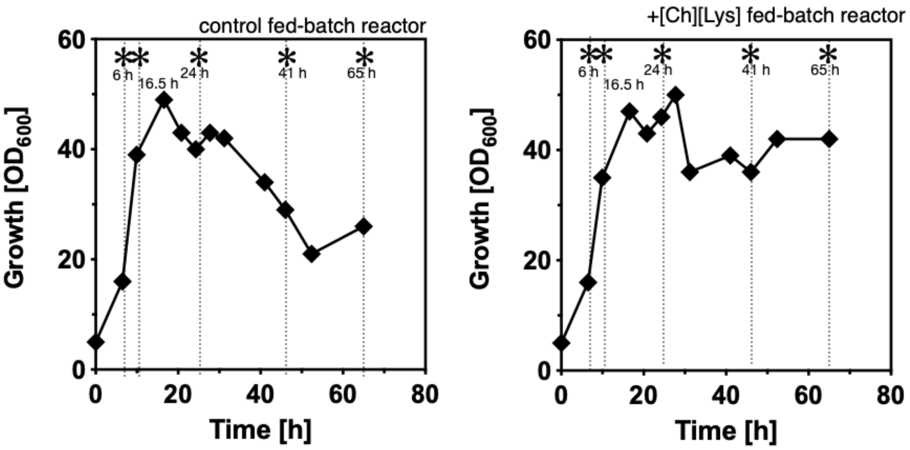

Figure S2. Offline OD_{600} measurements of isopentenol producing *C. glutamicum* grown with or without [Ch][Lys] treatment.

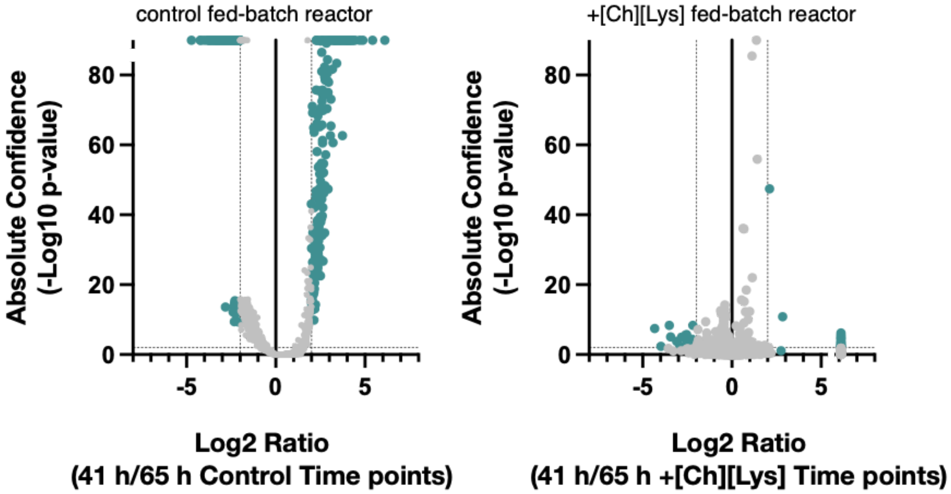

Figure S3. Differential gene expression analysis of *C. glutamicum* cells sampled from the same bioreactor nearing termination of fed batch campaign (41 hours / 65 hours) with or without exogenous [Ch][Lys] in culture media.

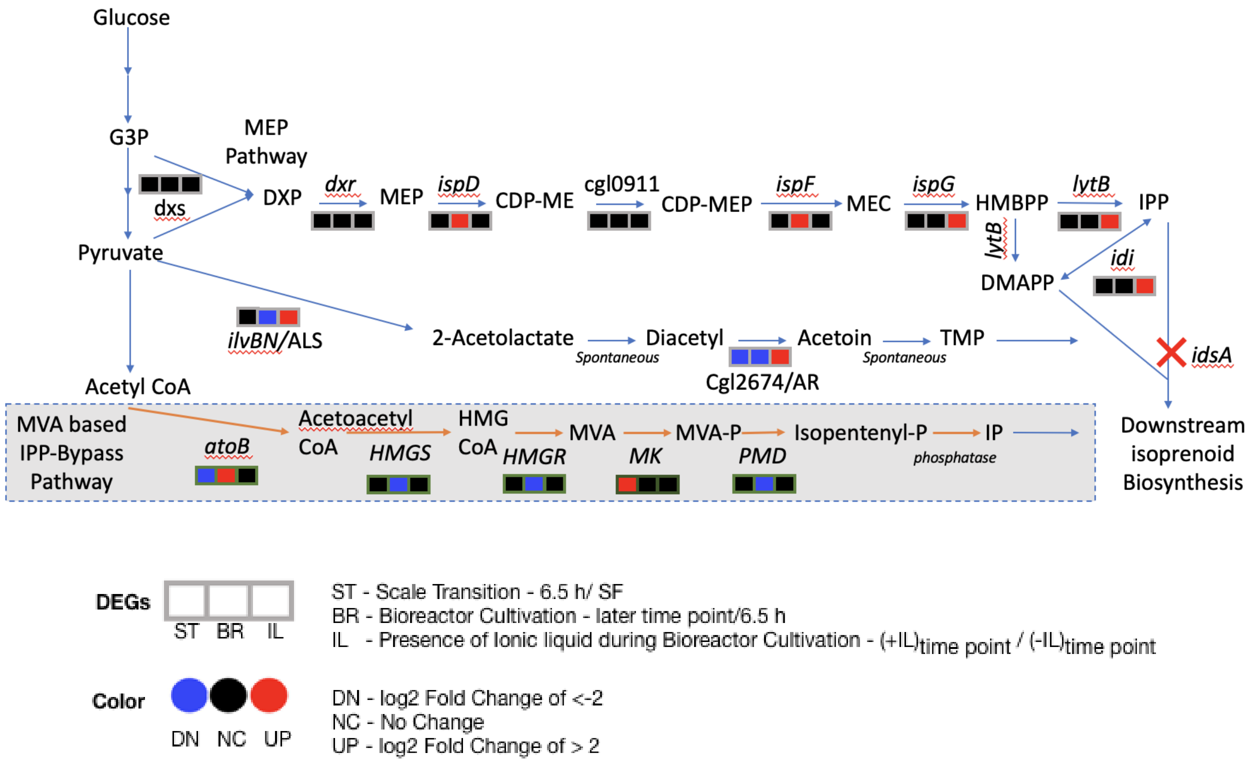

Figure S4: Differential transcript profiles of engineered *C. glutamicum* for isoprenoid biosynthesis and acetoin production. The native methylerythritol-4-phosphate (MEP) pathway is compared to the heterologous mevalonate (MVA) based isopentyl diphosphate (IPP) bypass pathway which results in isopentenol (IP) production. Three DEGs corresponding to three discrete conditions that were analyzed are represented here: ST - scale transition from shake flask (SF) to early bioreactor cultivation (6.5 h), BR - bioreactor later stage cultivation in the absence of IL and IL - bioreactor cultivation in the presence of IL compared to in the absence of IL. The heterologous pathway for IP production is shown in orange. Red crosses show the gene deletions in the *C. glutamicum* strain used in this study.

Abbreviations: *atoB*, acetyl-CoA acetyltransferase; CDP-ME, 4-(cytidine 5'-diphospho)-2-C-methyl-D-erythritol; CDP-MEP, 2-phospho-4-(cytidine 5'-diphospho)-2-C-methyl-D-erythritol; DMAPP, Dimethylallyl diphosphate; DXP, 1-deoxy-D-xylulose 5-phosphate; G3P, Glyceraldehyde 3-phosphate; HMBPP, 1-hydroxy-2-methyl-2-(E)-butenyl 4-diphosphate; *HMGS*, hydroxymethylglutaryl-CoA synthase; *HMGR*, 3-hydroxy-3-methylglutaryl-CoA reductase; HMG CoA, 3-hydroxy-3-methyl-glutaryl-coenzyme A; IP, Isopentenol; IPP, Isopentenyl diphosphate; MEC, 2-C-methyl-D-erythritol 2,4-cyclodiphosphate; MEP, 2-C-methyl-D-erythritol 4-phosphate, *MK*, mevalonate kinase; MVA, mevalonate; MVA-P, mevalonate; PMD, phosphomevalonate decarboxylase; TMP, tetramethylpyrazine.

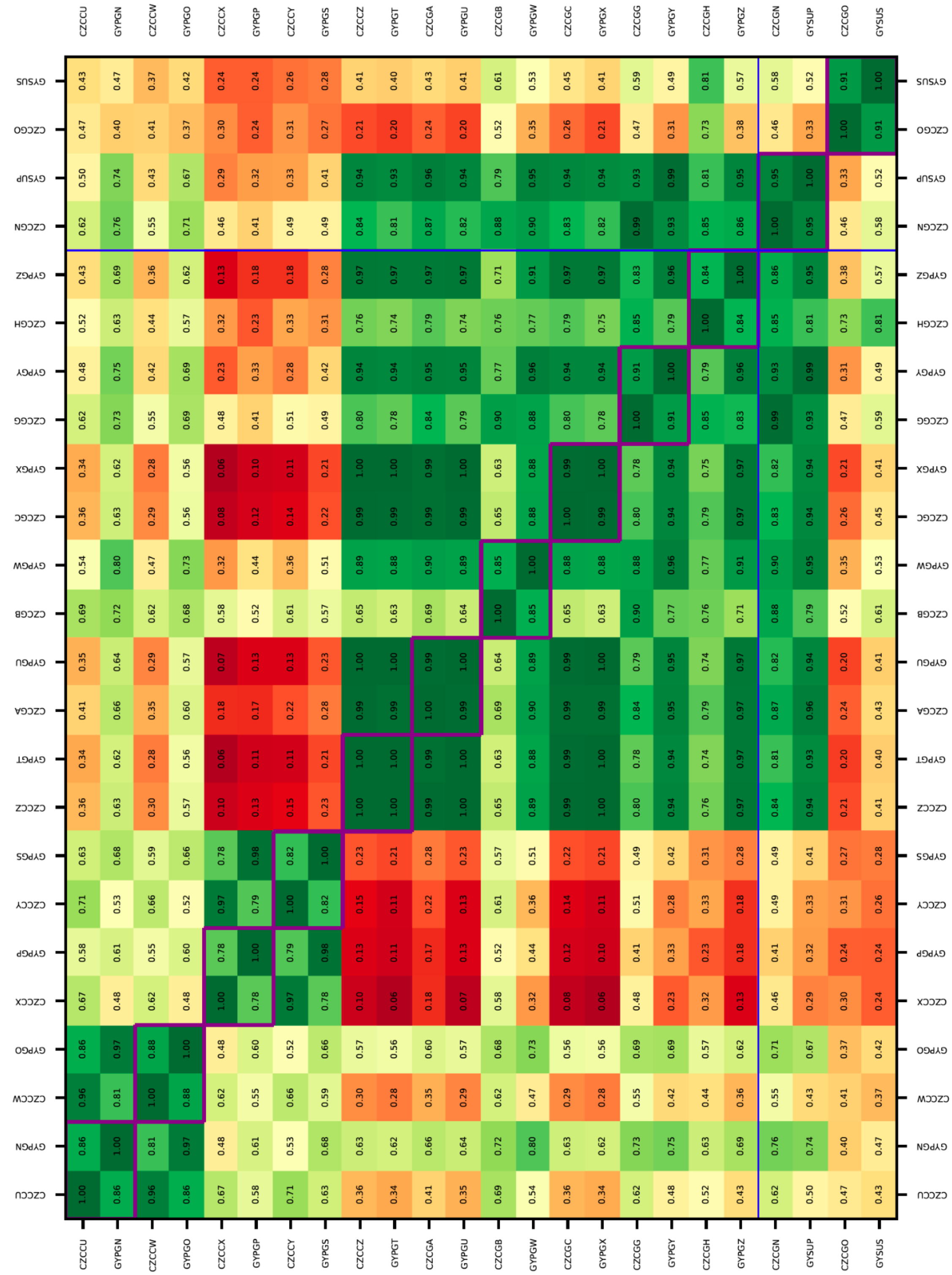

Figure S5: Raw gene counts were used to evaluate the level of correlation between biological replicates using Pearson's correlation. Refer to Table S3 for the identity of the sample libraries.

Table S1: A comparison of the average nucleotide identity of the twenty eight identified *Corynebacterium glutamicum* isolates with sequence information.

JGI IMG Genome ID	Strain Name	JGI IMG Genome ID	Strain Name	Average Nucleotide Identity (%)
2821586876	*C. glutamicum* BRC-JBEI 1.1.2	2541047465	*C. glutamicum* Z188	99.9987
2821586876	*C. glutamicum* BRC-JBEI 1.1.2	2554235421	*C. glutamicum* SCgG1	99.9987
2821586876	*C. glutamicum* BRC-JBEI 1.1.2	2554235422	*C. glutamicum* SCgG2	99.9987
2821586876	*C. glutamicum* BRC-JBEI 1.1.2	2534681606	*C. glutamicum* S9114	99.9979
2821586876	*C. glutamicum* BRC-JBEI 1.1.2	2545824695	*C. crenatum* MT	99.9755
2821586876	*C. glutamicum* BRC-JBEI 1.1.2	2585428016	*C. crenatum* SYPA 5-5	99.9718
2821586876	*C. glutamicum* BRC-JBEI 1.1.2	640427110	*C. glutamicum* R	98.9345
2821586876	*C. glutamicum* BRC-JBEI 1.1.2	2636415695	*Brevibacterium flavum* ATCC 15168	97.8554
2821586876	*C. glutamicum* BRC-JBEI 1.1.2	2848153583	*C. glutamicum* ATCC 14067	97.8528
2821586876	*C. glutamicum* BRC-JBEI 1.1.2	2687453329	*C. glutamicum* YI	97.8298
2821586876	*C. glutamicum* BRC-JBEI 1.1.2	2531839299	*C. glutamicum* ATCC 14067	97.8291
2821586876	*C. glutamicum* BRC-JBEI 1.1.2	2654587970	*C. glutamicum* B253	97.8238
2821586876	*C. glutamicum* BRC-JBEI 1.1.2	2869752021	*C. glutamicum* AJ1511	97.7627
2821586876	*C. glutamicum* BRC-JBEI 1.1.2	2859560989	*C. glutamicum* WM001	97.7553
2821586876	*C. glutamicum*	2718218481	*C. glutamicum*	97.7534

	BRC-JBEI 1.1.2		ATCC 13869	
2821586876	*C. glutamicum* BRC-JBEI 1.1.2	2718217803	*C. glutamicum* XV	97.7402
2821586876	*C. glutamicum* BRC-JBEI 1.1.2	2684623129	*C. glutamicum* CP	97.7373
2821586876	*C. glutamicum* BRC-JBEI 1.1.2	2859553545	*C. glutamicum* B414	97.7355
2821586876	*C. glutamicum* BRC-JBEI 1.1.2	2802429473	*C. flavum* ZL-1	97.7246
2821586876	*C. glutamicum* BRC-JBEI 1.1.2	2718218485	*C. glutamicum* ATCC 21831	97.6605
2821586876	*C. glutamicum* BRC-JBEI 1.1.2	2850571192	*C. glutamicum* AR1	97.6587
2821586876	*C. glutamicum* BRC-JBEI 1.1.2	2823997243	*C. glutamicum* ATCC 13287	97.6561
2821586876	*C. glutamicum* BRC-JBEI 1.1.2	2859564202	*C. glutamicum* ATCC 13032	97.6535
2821586876	*C. glutamicum* BRC-JBEI 1.1.2	639279306	*C. glutamicum* Kalinowski ATCC 13032	97.6478
2821586876	*C. glutamicum* BRC-JBEI 1.1.2	2554235405	*C. glutamicum* MB001	97.6418
2821586876	*C. glutamicum* BRC-JBEI 1.1.2	639279307	*C. glutamicum* Nakagawa ATCC 13032	97.6392
2821586876	*C. glutamicum* BRC-JBEI 1.1.2	2554235360	*C. glutamicum* K051	97.6305
2821586876	*C. glutamicum* BRC-JBEI 1.1.2	2687453317	*C. glutamicum* USDA-ARS-USMA RC-56828	97.3362

Table S2: Strain and Plasmids Used in this Study.

Strain/Plasmid Name	Genotype	Reference
JBEI-18084	*C. glutamicum* ATCC 13032 Δ*cglIM* Δ*cgLIR* Δ*cgLIIR* biotin auxotroph, NxR.	A kind gift from Sarah Richardson. Described in Baumgart *AEM*, 2013. [Sequenced at JGI. ProjectID: 503247]
JBEI-7936	*C. glutamicum* BRC-JBEI 1.1.2 biotin auxotroph, NxR	Sasaki and Eng *et al,* BfB, 2019. [Sequenced at JGI. ProjectID: Ga0373873]
JBEI-19566	*C. glutamicum* BRC-JBEI 1.1.2 Δ*ldh* Δ*idsA* biotin auxotroph, NxR derived from JBEI-7936	Sasaki and Eng *et al,* Met Eng Comm. [Sequenced at JGI. Project ID: 503805]
JBEI-19559	*E. coli* DH10B {pEC-XK99E-AK-IP-bypass} KanR	Sasaki and Eng *et al,* BfB, 2019.
JBEI-19632	*E. coli* DH10B {pEC-XK99E-AK-IP-bypass-*S. pomeroyi* HMGR (*substitution*)} KanR	Sasaki and Eng *et al,* BfB, 2019.

Table S3: RNAseq Sample key and library QC metrics for samples analyzed in the fed-batch campaign.

Library Name	Sample Id	Raw Reads	Filtered Reads	Sample Name
CZCCU	191641	6565438	2547302	shake flask / final seed train culture CGXII + 4% glucose
GYPGN	191641	3783644	3447814	shake flask / final seed train culture CGXII + 4% glucose
CZCCW	191642	10201048	4842164	shake flask / final seed train culture CGXII + 4% glucose
GYPGO	191642	8921860	8194336	shake flask / final seed train culture CGXII + 4% glucose
CZCCX	191643	9942954	1265252	6.5 hrs post innoc 2 L fed-batch CGXII + 50 mM [Chl][Lys]
GYPGP	191643	8646938	8023706	6.5 hrs post innoc 2 L fed-batch CGXII + 50 mM [Chl][Lys]
CZCCY	191644	17316250	3273128	6.5 hrs post innoc 2 L fed-batch CGXII
GYPGS	191644	6786960	6327830	6.5 hrs post innoc 2 L fed-batch CGXII
CZCCZ	191645	10710608	7635578	16.58 hrs post innoc 2 L fed-batch CGXII + 50 mM [Chl][Lys]
GYPGT	191645	7781964	5910924	16.58 hrs post innoc 2 L fed-batch CGXII + 50 mM [Chl][Lys]
CZCGA	191646	5558460	499094	16.58 hrs post innoc 2 L fed-batch CGXII
GYPGU	191646	5876548	4501932	16.58 hrs post innoc 2 L fed-batch CGXII
CZCGB	191647	8645056	8167270	24 hrs post innoc 2 L fed-batch CGXII 4% + 50 mM [Chl][Lys]
GYPGW	191647	8696772	7905458	24 hrs post innoc 2 L fed-batch CGXII 4% + 50 mM [Chl][Lys]
CZCGC	191648	12856598	5616520	24 hrs post innoc 2 L fed-batch CGXII
GYPGX	191648	6564560	5652508	24 hrs post innoc 2 L fed-batch CGXII
CZCGG	191649	8383158	7336994	41 hrs post innoc 2 L fed-batch CGXII + 50 mM [Chl][Lys]
GYPGY	191649	8162496	7448506	41 hrs post innoc 2 L fed-batch CGXII + 50 mM [Chl][Lys]
CZCGH	191650	6516938	784488	41 hrs post innoc 2 L fed-batch CGXII
GYPGZ	191650	7872554	6521852	41 hrs post innoc 2 L fed-batch CGXII
CZCGN	191651	6702200	868060	65 hrs post innoc 2 L fed-batch CGXII + 50 mM [Chl][Lys]
GYSUP	191651	7061322	310186	65 hrs post innoc 2 L fed-batch CGXII + 50 mM [Chl][Lys]
CZCGO	191652	19711092	1380996	65 hrs post innoc 2 L fed-batch CGXII
GYSUS	191652	7430190	90368	65 hrs post innoc 2 L fed-batch CGXII
